# Adhesive capsulitis: the importance of early diagnosis and treatment

**DOI:** 10.1007/s40477-024-00891-y

**Published:** 2024-06-06

**Authors:** Fabio Vita, Davide Pederiva, Roberto Tedeschi, Paolo Spinnato, Flavio Origlio, Cesare Faldini, Marco Miceli, Salvatore Massimo Stella, Stefano Galletti, Marco Cavallo, Federico Pilla, Danilo Donati

**Affiliations:** 1 Department of Orthopedic and Traumatological Surgery, IRCCS Istituto Ortopedico Rizzoli, University of Bologna, Bologna, Italy; 2 Department of Biomedical and Neuromotor Sciences, Alma Mater Studiorum, University of Bologna, Bologna, Italy; 3 Diagnostic and Interventional Radiology, IRCCS Istituto Ortopedico Rizzoli, Bologna, Italy; 4 Physical Therapy and Rehabilitation Unit, IRCCS Istituto Ortopedico Rizzoli, University of Bologna, Bologna, Italy; 5 SIUMB Advanced School for Musculoskeletal Ultrasound, Department of Clinical and Experimental Medicine, University Post-Graduate Course, Santa Chiara University Hospital, Pisa, Italy; 6 Musculoskeletal Ultrasound School, Italian Society for Ultrasound in Medicine and Biology, Bologna, Italy; 7 Department of Shoulder and Elobow UnitIRCCS Istituto Ortopedico Rizzoli, University of Bologna, Bologna, Italy; 8 Physical Therapy and Rehabilitation Unit, Policlinico Universitario di Modena, Modena, Italy; 9 Clinical and Experimental Medicine PhD Program, University of Modena and Reggio Emilia, Modena, Italy

**Keywords:** Adhesive capsulitis, Ultrasound, Early diagnosis, Hydrodistention, Rehabilitation

## Abstract

**Background:**

Adhesive capsulitis (AC), more commonly known as "frozen shoulder”, is a painful shoulder condition. The illness progresses through three phases: freezing, frozen and thawing. A gold standard treatment for adhesive capsulitis is not defined. The goal of any treatment is to reduce pain and restore shoulder movement.

**Objective:**

Objective of the present study is to evaluate the efficacy of gleno-humeral hydrodistension associated with physical therapy in patients with diagnosed adhesive capsulitis comparing the outcomes in term of pain and range of motion in patients with a phase 1 and a phase 2 disease.

**Method:**

Between January 2022 and April 2023, We evaluated 87 patients with adhesive capsulitis, 47 were excluded for others concomitant pathologies, finally 40 patients were enrolled for the study, of whom 23 had capsulitis in stage 1 and 17 in stage 2. Patients were evaluated at baseline and at 2, 4 and 6 months after infiltration recording range of motion in all planes, pain and functionality scores.

**Results:**

A significant improvement was recorded in shoulder range of motion in all planes with the except of extension in both groups. Phase 2 patients were able to regain shoulder range of motion in all planes except internal rotation which was recovered with more difficulty. Pain and functionality scores improved significantly between baseline and follow-up visits.

**Conclusion:**

Ultrasound-assisted hydrodistention of the glenohumeral joint combined with targeted exercise has been successful in improving pain relief, reducing disability, and increasing range of motion in subjects with stage 1 and 2 adhesive capsulitis, especially if diagnosed before phase 2 (when the range of motion is completely reduced).

## Introduction

Adhesive capsulitis (AC), more commonly known as "frozen shoulder”, is a painful shoulder condition. The terminology was first used by Codman in 1934 and later modified by Neviaser in 1945, who introduced the term "adhesive capsulitis" to describe a change in the glenohumeral joint synovium [1]. Symptoms of this condition include progressive loss of range of motion (ROM) in the shoulder joint, with external rotation being most affected. The current diagnostic consensus definition points out that AC is a condition characterized by significant reduction of both active and passive shoulder motion that occurs in the absence of a known intrinsic shoulder disorder. This condition, that has a higher incidence in women between 40 and 60 years of age [2], results from progressive fibrosis and contracture of the glenohumeral joint capsule, which causes pain and stiffness [1]. Despite advances in its understanding, the exact pathophysiology of this condition remains unclear, and its natural history and recovery time remain difficult to evaluate.

Adhesive capsulitis can be classified as primary and secondary [3].

Primary adhesive capsulitis is idiopathic and is characterized by a gradual and painful restriction in the shoulder's active and passive motion. Secondary adhesive capsulitis has similar symptoms but has an identifiable cause [3]. It is also important to note that certain conditions such as diabetes, calcific tendinopathy of the rotator cuff tendons during the painful resorptive phase, autoimmune thyroid diseases, Parkinson's disease and other neurologic conditions, cardiac issues or autoimmune diseases can increase the risk of developing adhesive capsulitis [4].

The illness progresses through three phases: the "freezing" phase, which lasts 2 to 9 months with increased pain and decreased movement; the "frozen" phase, which endures between 4 to 12 months and is characterized by decreasing pain but persistent stiffness; and the "thawing" phase, when recovery begins with a gradual enhancement in range of motion, lasting 12–24 months [5].

Diagnosing AC is done through a combination of clinical examination and imaging. It is essential to exclude other potential causes of shoulder pain and stiffness, such as septic arthritis, fractures, rotator cuff issues, and cervical radiculopathy, prior to diagnosing adhesive capsulitis. In the early stages, pain and stiffness are the most common indicators, typically located on the anterolateral side of the shoulder, the anterior and middle of the upper arm, and sometimes on the flexor surface of the forearm. Pain can be especially prominent at night [5].

There are physical signs associated with AC, such as decreased muscle strength and mobility of the deltoid and supraspinatus muscles, and a reduced angle between the humerus and scapula [4]. X-rays are not usually helpful except to exclude bone pathologies, but magnetic resonance (MRI) is the most reliable imaging method. Ultrasound image is also a popular choice since it is inexpensive, accessible, and can differentiate it from other conditions [6, 7].

Specific signs of adhesive capsulitis on ultrasound evaluation include thickening of the inferior recess of the glenohumeral joint capsule, thickening of the coracohumeral ligament and soft tissue structures in the rotator cuff interval, hypervascularization of the subacromial-deltoid bursa, an effusion of the biceps tendon sheath [6–8] and a typical folding of the infraspinatus tendon during posterior assessment of passive external rotation during maneuvers [4].

Few studies have investigated the relationship between clinical phases and ultrasound findings. Zappia et al. [9] showed that rotator interval was thicker in phase 1 than in phase 2, and the coracohumeral ligament was significantly thicker in phase 1 compared to normal. Additionally, thickened synovium and synovial proliferation with adhesion in the rotator interval have been observed in phase 2 [8]. But also thickening of axillar pouch, according with our practical observations, is seen in phase 1 and 2 [2]. This suggests that synovial inflammation and proliferation affects the thickness of the rotator interval and coracohumeral ligament in the second phase of the disease.

The goal of treatment is to reduce pain and restore shoulder movement. Physical therapy, manipulation under anesthesia, ultrasound-guided capsule distention, subacromial injection, arthroscopic capsule release, and open surgery have all been described as treatment options for AC [1, 10, 11].

Hydrodistension is a technique that involves injecting a combination of saline, corticosteroids, and anesthetic into the shoulder joint, which can increase the hydrostatic pressure and volume capacity of the joint, providing quick relief [12, 13]. Injection with ultrasound guidance has been shown to be more reliable than injection without guidance [14] and faster than fluoroscopic-guided injection [1] and is therefore the preferred method [13]. A cochrane review [15] has shown that hydrodistension with saline and steroids in patient with a diagnosis of frozen shoulder reduces pain after 3 weeks and disability after 12 weeks. Ladermaan et al. recently confirmed this conclusion [16].

Associated to the infiltrative treatment, physiotherapy is gaining more and more importance. Physiotherapist-led interventions usually consist of patient education and joint passive and active mobilization. Exercise therapy has been proven to be effective in reducing pain and disability in several shoulder conditions, and usually is part of a multi-modal program [17]. The most common types of exercises are isometric or strengthening exercises of rotator cuff, trapezius, scapular, and glenohumeral muscles, Codman pendulum exercises and stretching exercises [10, 18, 19]. The aim of these exercises is to improve range of motion and muscle function by restoring shoulder mobility and stability.

A lack of consensus of the phase-appropriate suggested intervention is lacking [5]. The objective of the present study is to underline the importance of early diagnosis in patients with adhesive capsulitis, highlighting how a late diagnosis with the transition from phase 1 to phase 2, can lead to a prolongation of physiotherapy treatments and infiltrative.

## Materials and methods

A prospective evaluation of the patients with a diagnosis of adhesive capsulitis between September 1st 2022 and April 28th 2023 was performed. The patients were evaluated clinically and radiographically (anteroposterior and axillary shoulder X-ray) to exclude other possible pathologies with similar symptoms (e.g. calcific tendinopathy in resorptive phase, bursitis SAD, glenohumeral osteoarthritis, etc.) and the final diagnosis was given by a senior orthopedic surgeon specialized in shoulder pathology. The exclusion criteria were: pregnancy, reported allergies to anesthetics, diagnosis other than adhesive capsulitis, patients unable to give consent.

We evaluated 87 patients with adhesive capsulitis, 47 patients were excluded as they did not present primary adhesive capsulitis, due to the presence of other concomitant pathologies (11 for glenohumeral arthrosis, 7 for complete rotator cuff rupture, 8 had bursal effusions, 4 neurological pathologies, 8 had recent fractures upper limbs, 9 had calcification of the supraspinatus in the reabsorption phase), finally 40 patients were enrolled in the study [20], finally 40 patients were enrolled for the study, of whom 23 had capsulitis in stage 1 and 17 in stage 2.

Each patient underwent ultrasound and clinical evaluation, performed by an experienced sonographer in musculoskeletal ultrasound and an orthopedic surgeon specialized in upper extremity surgery, respectively. A physical therapist, lastly, performed his evaluation.

In each patient the following data was recorded: sex, age, Visual Analogue Scale (VAS) pain, duration of symptoms, shoulder range of motion in all planes (flexion, extension, abduction, external and internal rotation). For each patient the following scores were calculated: Disability of the Arm, Shoulder and Hand (DASH) [21], Shoulder Pain and Disability Index (SPADI) [22] and American Shoulder and Elbow Surgeons (ASES) [23].

Patients were divided into 2 groups based on the clinical range of motion of the shoulder and the duration of symptoms [5].

### Ultrasound diagnosis

The ultrasound examinations of the shoulder were performed using an Sonoscape X3 Pro with a 5–17 MHz linear transducer with musculoskeletal preset.

**Fig. 1:**
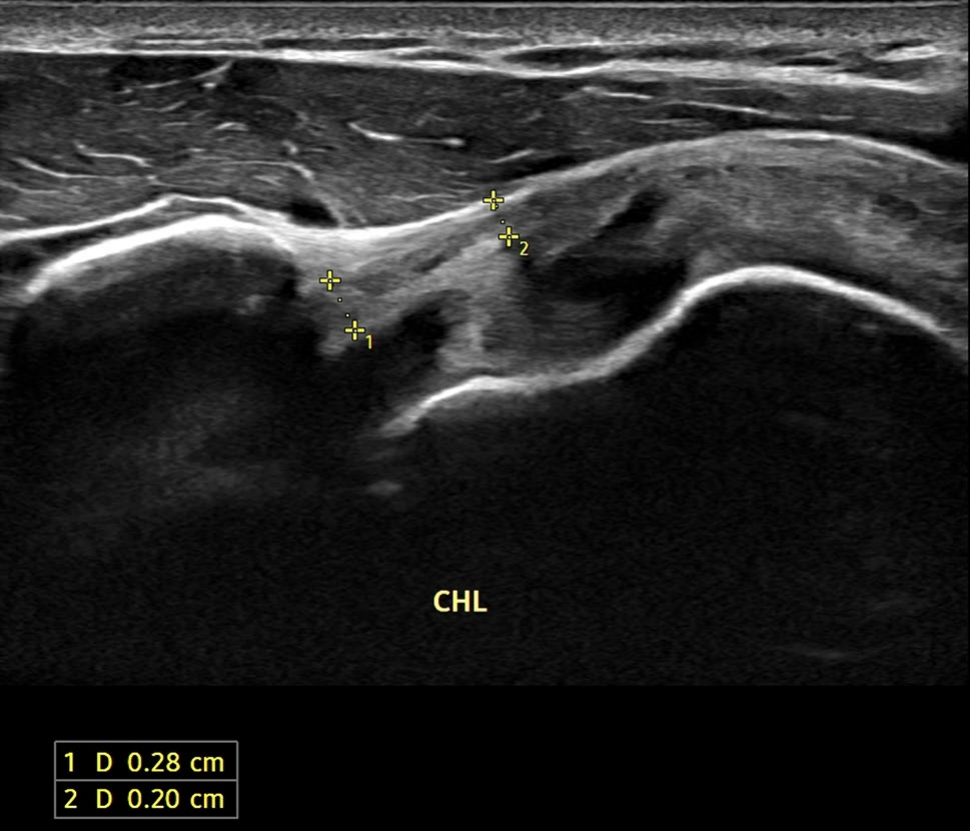
Thickening of the coraco-humeral ligament

**Fig. 2:**
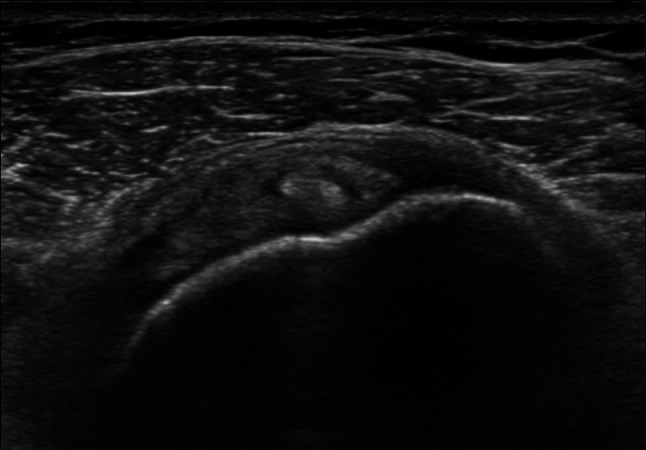
Thickening of rotator interval (RI)

**Fig. 3:**
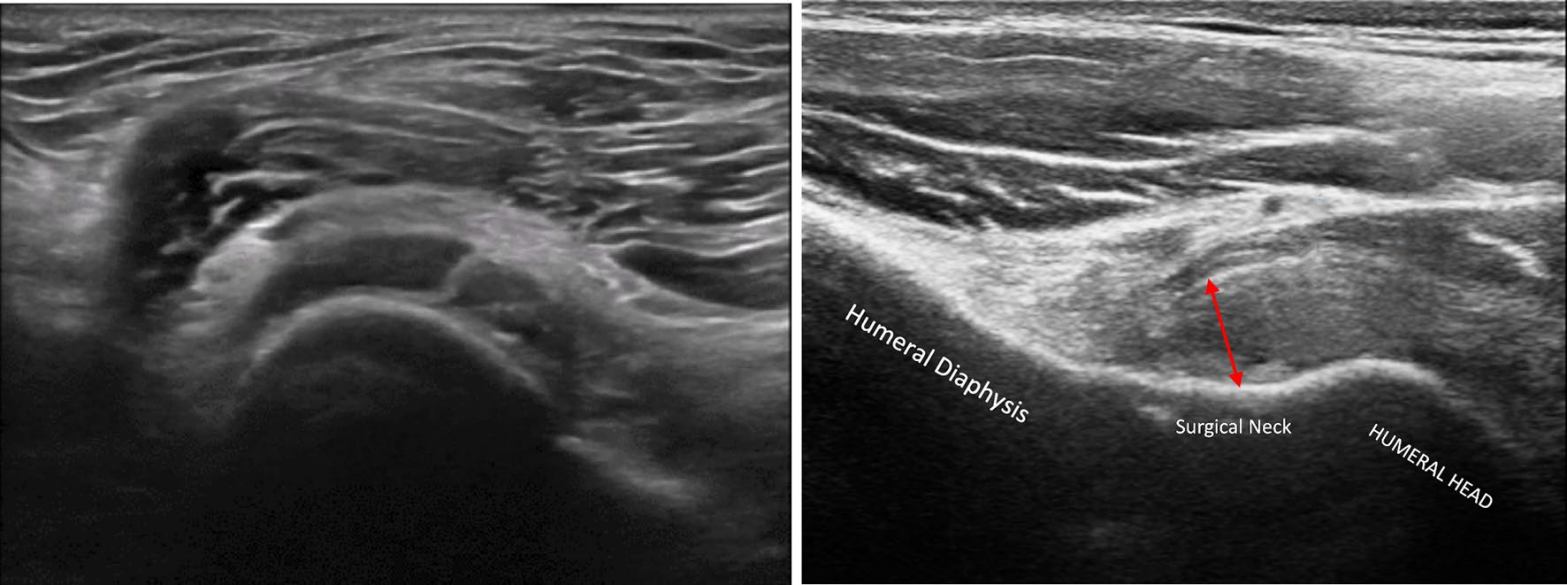
Thickening of the inferior recess of the glenohumeral joint capsule (axially on the left and longitudinally on the right)

**Fig. 4:**
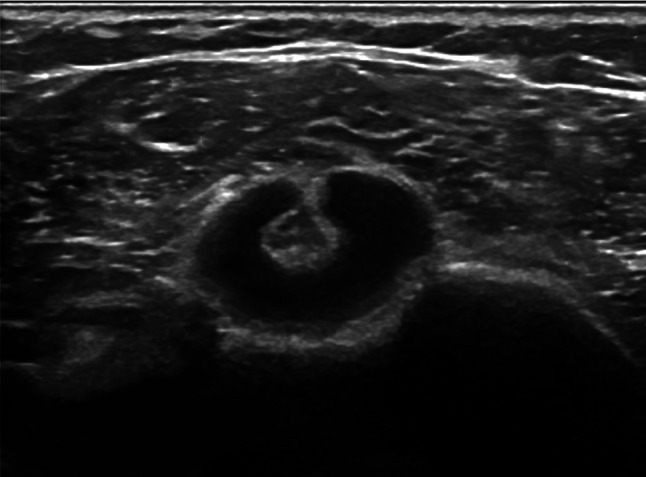
Long head of the biceps tendon (LHBT) sheath effusion

**Fig. 5:**
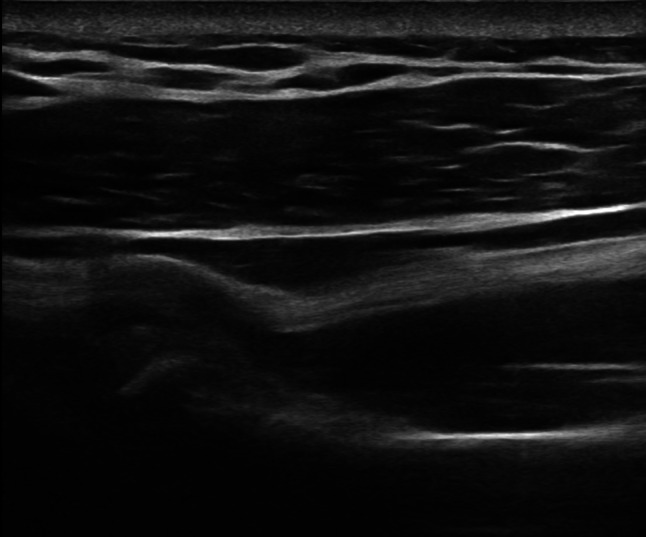
The folding of the infraspinatus tendon during passive external rotation

The following parameters were assessed:

- Thickening of coracohumeral ligament (CHL): patients were scanned in a sitting position, with the shoulder in a neutral position and the hand resting on their thigh, by positioning the transducer on the lateral border of the coracoid process in an axial oblique plane, obtaining a longitudinal image of the CHL (Fig. 1).
- Thickening of rotator interval (RI) and hypervascularization: The RI is formed superiorly by the anterior aspect of the supraspinatus tendon and inferiorly by the superior surface of the subscapularis tendon, and the medial border is formed by the lateral edge of the coracoid process, is evaluated in the plane of the oblique axis with the patient's fist held laterally in a sitting position. For RI evaluation for hypervascularization, B-mode ultrasound and power Doppler were performed (PRF 0.8 kHz). RI thickness was measured as the shortest distance between the long head of the biceps tendon and the peribursal fat, including the CHL, the superior glenohumeral ligament (Fig. 2).
- Thickening AR in the anterior/axillary regions: limited motion and increased thickness of the anterior shoulder capsule could potentially limit the range of motion of the subscapular muscle–tendon unit during active rotations of the glenohumeral joint. The inferior recess of the capsule is measured on a transverse plane and the value obtained is compared with that of the asymptomatic contralateral shoulder (Fig. 3).
- LHBT sheath effusion: The synovial sheath surrounding the long head of the biceps tendon (LHBT) typically connects with the glenohumeral joint cavity. Usually, in regular conditions, a small amount of fluid is detectable around the biceps which tends towards an eccentric position. Limitation of active and passive movements may also result from entrapment of the LHBT within the rotator range. This entrapment is due to the formation of capsular fibrosis or focal synovitis around the tendon sheath. It therefore becomes essential to perform a dynamic evaluation of the rotator interval, observing the real-time interactions between the tendon and the stabilizing pulley (Fig. 4).
- The folding of the infraspinatus tendon during passive external rotation (Fig. 5): the posterior dynamic study during a passive external rotation shows reduced sliding with the tendon folding towards the joint capsule (its profile changes from flat to concave). This behavior in often associated with a ‘bouncing’ movement of the tendon which returns to its baseline resting position after a little jolt.

### Ultrasound-guided infiltrative treatment

**Fig. 6:**
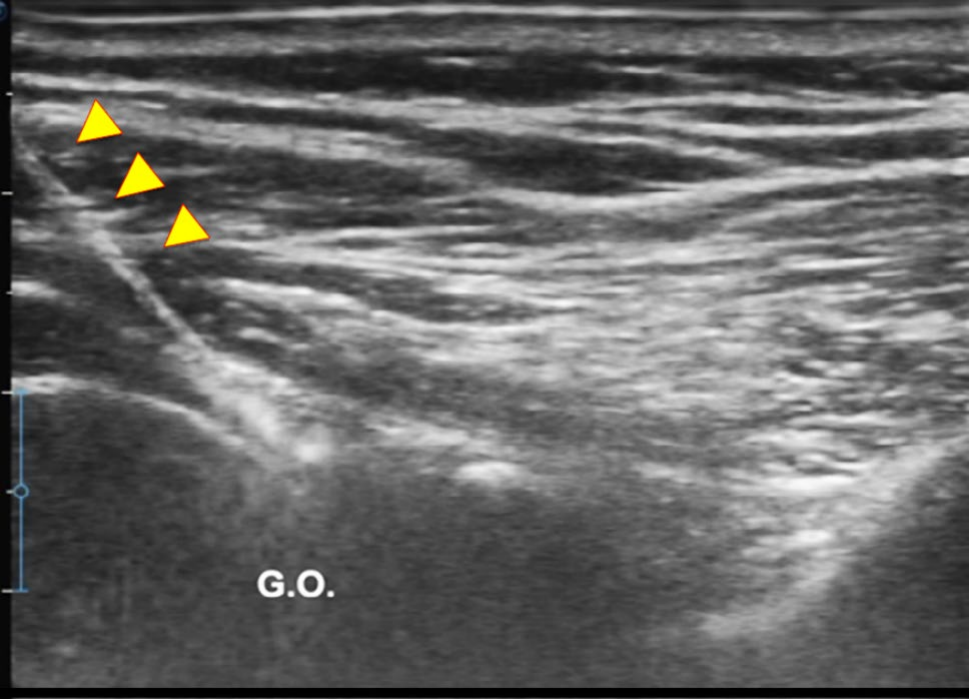
Ultrasound-guided infiltrative treatment: hydrodistention of the gleno-humeral joint through a posterior access to the shoulder joint

All patients were treated with one infiltrative hydrodistension procedure under ultrasound guidance, performed by an experienced sonographer in musculoskeletal ultrasound. All injections for glenohumeral joint were performed with the patient in a prone position, using a 90 mm long 20 G needle, after disinfecting the skin with a solution of povidone iodine or chlorhexidine, through a latero to medial posterior access to the shoulder joint (Fig. 6). 1 ml cortisone (depomedrol 40 mg), 10 ml of 2% lidocaine hydrochloride and 10 ml of saline were injected [12, 13].

### Rehabilitation treatment

Immediately after the infiltration, the patients were referred to the Department of Physics and Rehabilitation to begin rehabilitation treatment.

The rehabilitation protocol consisted of a series of pendulum exercises and passive/active glenohumeral mobilization exercises (external and internal rotation, front elevation and retroposition).

**Fig. 7:**
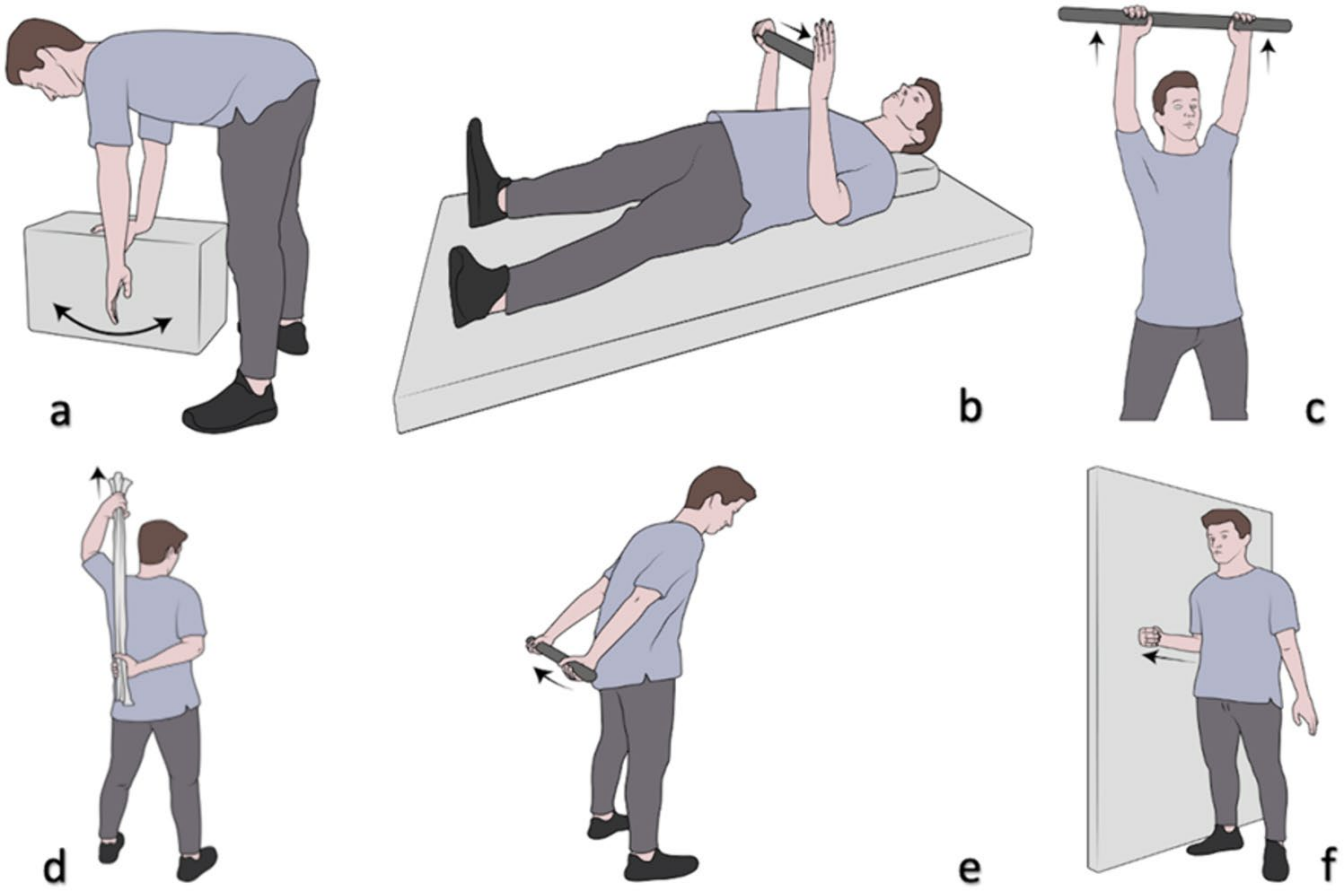
**a**–**f** Physiotherapy exercises with personal rehabilitation protocol (see text for details)

Each patient was instructed to perform these exercises twice daily for 15 min per session and the exercises were:

*Commuting exercises* The patient bends the torso forward so that it is parallel to the floor and leans on a stool or table with the sound arm. They swing the affected limb back and forth for about 5 min. With the treated limb, the patient makes circles out-wards with the palm facing out, and then circles inwards with the palm (Fig. 7a).

*External rotation exercises* The patient is supine, with the arm close to the body, and the elbow flexed at 90°. The patient holds a stick with the healthy limb and places it on the palm of the affected limb, pushing on it so as to hyper-rotate the affected limb. The patient maintains this position for about 15–20 s and then returns to the starting position (Fig. 7b).

*Anterior elevation exercises* Standing upright, the patient holds a cane with both hands, raises their arms above their head as far as they can, holding the position for about 10 s and then returns to the starting position (Fig. 7c).

*Internal rotation exercises* The patient places the treated limb behind their back with the elbow bent. Using a towel and using the healthy limb, they bring the treated limb to the maximum internal rotation, maintaining this position for about 5 s (Fig. 7d).

*Retroposition exercises* Standing upright, the patient grasps a stick behind their back with both hands and brings their shoulders to the maximum retroposition, maintaining this position for 5 s (Fig. 7e).

*Abduction exercises* Standing next to a wall with the elbow flexed at 90°, the pa-tient pushes their elbow and forearm against the wall (Fig. 7f).

Follow-up visits were scheduled after 2, 4 and 6 months. At each follow-up visit a US scan was performed looking for the diagnostic criteria listed above. The same orthopedic surgeon performed a clinical evaluation, and the following were recorded: VAS pain, shoulder range of motion in all planes, DASH score, SPADI score and ASES score.

### Statistical analysis

All continuous variables will be expressed in terms of mean ± standard deviation (SD) and range. Categorical variables will be summarized in terms of absolute frequency and percentage. Correlations between means of outcome measures pre-treatment and post-treatment were analyzed with *T*-test; 0.05 was considered significant. The statistical analysis was performed using the statistical package for social sciences (SPSS), software version 15.0 (SPSS Inc., 199 Chicago, USA) by a statistical consultant from Rizzoli Orthopedic Institute.

## Results

Between September 1st 2022 and April 28th 2023 a total of 87 patients were diagnosed with adhesive capsulitis, of these 40 met the inclusion criteria. Of these 40 patients, 20 entered Phase 1 and 20 entered Phase 2. No patients were lost during the 6-month follow-up.

The first group consisted of 13 women and 10 men, with a mean age of 52 years (min 43–max 67) and a mean duration of symptoms of 4 months (min 1–max 9). The second group consisted of 10 women and 7 men, with a mean age of 53 years (min 42–max 70) and a mean duration of symptoms of 14 months (min 10–max 18).

Statistical analysis of shoulder mobility showed a statistically significant improvement in both group in all movements with exception of extension (Table 1).

**Table 1:** Shoulder ROM, expressed in degrees, in Phase 1 (P1) and Phase 2 (P2) at baseline and at each follow-up visit

	Baseline		2-months		4-months		6-months		*T*-test
	Mean	SD	Mean	SD	Mean	SD	Mean	SD	*p* value
**Flexion**									
Pl	122	22	139	21	152	25	156	23	< 0.0005
P2	77	18	113	34	143	28	154	22	< 0.0005
**Extension**									
Pl	37	9	47	9	48	9	48	8	0.624
P2	30	11	45	12	48	9	49	7	0.413
**Abduction**									
Pl	94	35	113	24	119	30	141	29	0.001
P2	64	30	87	35	109	35	130	22	< 0.0005
**Intra**									
Pl	38	23	55	18	52	22	63	19	0.004
P2	16	15	45	23	46	18	50	12	0.057
**Extra**									
Pl	43	29	62	26	64	21	70	21	< 0.0005
P2	11	12	37	23	44	23	58	12	< 0.0005

No adverse events were recorded during the procedure or during the follow-up.

In phase 1, the mean measurement of flexion before treatment was 122 with a standard deviation of 22 and in phase 2, the mean was 77 with a standard deviation of 18. At follow-up after six months in both phases of frozen shoulder the results showed statistically significant improvements (*p* < 0.0005) and clinically significant changes at all time points from baseline and between all time points, indicating a continued functional recovery of movement.

In both phases the results of the extension didn’t show statistically significant improvement.

In addition, significant improvements were seen between all time points for range of motion in abduction (phase 1, *p* = 0.001; phase 2, *p* < 0.0005) and extrarotation (*p* < 0.0005).

In intrarotation the result showed statistically significant improvement in the phase 1 of adhesive capsulitis (*p* = 0.004) but less in phase 2 (*p* = 0.057).

Statistical analysis of shoulder pain and functionality score showed a statistically significant improvement in both group in all the explored variables (Table 2).

**Table 2:** Shoulder VAS pain and functionality scores in Phase 1 (P1) and Phase 2 (P2) at baseline and at each follow-up visit

	Baseline		2-months		4-months		6-months		*T*-test
	Mean	SD	Mean	SD	Mean	SD	Mean	SD	*p* value
**VAS**									
Pl	7	2	3	2	1	2	1	2	< 0.0005
P2	5	2	2	2	1	2	1	2	< 0.0005
**DASH**									
Pl	39	16	20	15	11	9	10	10	0.624
P2	43	15	21	13	13	14	12	18	0.413
**SPADI**									
Pl	48	23	23	16	12	12	11	13	< 0.0005
P2	50	21	30	19	20	20	17	19	0.001
**ASES**									
Pl	46	23	27	16	15	IO	12	9	< 0.0005
P2	46	17	25	15	18	14	15	19	< 0.0005

Regarding the SPADI scale, before treatment patients with phase 1 of frozen shoulder had a mean score of 48% ± 23 and patients with phase 2 had a mean score of 50% ± 21. After the treatment the results showed marked improvement in both phases (phase 1, *p* < 0.0005; phase 2, *p* = 0.001).

Additionally, the values of the ASES scale and of the VAS at follow-up of six months showed marked and statistically improvement in both phases of adhesive capsulitis (*p* < 0.0005).

DASH results demonstrated a clinically significant sustained change at all three time points in both phases but didn’t showed statistically improvement at *T*-test (phase 1 *p* = 0.624, phase 2 *p* = 0.413).

All patients were treated with hydrodistention at baseline, 7 out of 23 patients in phase 1 were reinfiltrated after two months and 3 of these after another 2 months, while in phase 2, 14 out of 17 patients were reinfiltrated after 2 months, another 10 after 4 months and finally 4 patients were infiltrated 6 months after the first treatment (Table 3).

**Table 3:** Number of glenohumeral hydrodistentions in patients in Phase 1 (P1) and Phase 2 (P2) at baseline and at each follow-up visit

	Baseline	2-months	4-months	6-months
**N° hydrod**				
P 1	23	7	3	0
P2	17	14	10	4

## Discussion

Adhesive capsulitis, also known as “frozen shoulder,” remains an enigmatic and disabling disease that afflicts large numbers of individuals. It manifests as persistent pain associated with functional limitation of the shoulder [24]. The progression of this condition occurs through distinct phases, with phase 1 and phase 2 showing varying degrees of limitation and impairment [5] as presented by our data.

In the present study, we evaluated the effectiveness of ultrasound-guided infiltrative therapy by hydrodistension of the glenohumeral joint capsule [25]. The described procedure proved successful in significantly increasing shoulder articularity in all planes except extension, significantly reducing pain and significantly increasing shoulder function, described by SPADI and ASES scores (DASH although it described substantial improvement, did not reach statistical significance) [26]. Analysis of treatment in the two phases (phase 1 freezing and phase 2 frozen) showed similar effectiveness of therapy. ROM improved considerably in both groups following infiltrative treatment, which allowed a reduction in the differences between phase 1 and phase 2 at the first follow-up and an almost entirely elimination of differences at the second follow-up. Despite the treatment, however, a persistent reduction in intra and extra rotation was found in phase 2 patients which led all these patients to be treated several times with hydrodistension. This data underlines the importance of early diagnosis in order to provide the patient with the best outcome.

When comparing treatment options, it has been observed that intra-articular corticosteroid injections offer quicker relief from symptoms compared to physiotherapy. However, the effectiveness of these injections is short-lived, lasting less than 6 weeks [27]. Interestingly, when a physiotherapy program is implemented following corticosteroid injections into the glenohumeral joints, a statistically significant improvement is observed [28].

Bryant et al. evaluated the effectiveness of ultrasound-assisted hydrodistension (with 10 ml of 1% lidocaine followed by 40 mg of triamcinolone acetonide and then 20 ml of 0.9% NaCl) with a posterior approach, followed by physiotherapeutic exercise guided, in patients with adhesive capsulitis [29].They documented a significant and sustained increase in SPADI scores, a significant increase in the quick DASH scores and a clinically significant increase in external rotation, flexion, and abduction, all of which were compared to the baseline at 6 weeks, 3 months, and 6 months. They failed to assess the effectiveness of phase 1 treatment relative to phase 2 treatment.

The mechanism of improvement in function and pain with hydrodistension in adhesive capsulitis remains unclear. Intrinsic and extrinsic factors may contribute to the pathophysiology of adhesive capsulitis.

### Limitations of the study

This study certainly has its limitations. The small sample size may be a cause of bias in the results obtained. The procedure is operator dependent, and this may result in difficulties in the reproducibility of the results. In addition, the criteria used in the division between phase 1 and phase 2 does not systematically respect the pathophysiological process of each patient, thus introducing a possible bias that future studies with increased sample numbers may go to verify. On the other hand, this study has aspects that make it noteworthy. The prospective nature and the division of patients according to disease stages make the study the first of its kind.

## Conclusion

Ultrasound-assisted hydrodistention of the glenohumeral joint combined with targeted exercises has been successful in improving pain relief, reducing disability, and increasing range of motion in subjects with stages 1 and 2 adhesive capsulitis. In particular, patients diagnosed with stage 1 adhesive capsulitis have better short-term results and faster recovery of full shoulder mobility, do not require numerous infiltrations and achieve clinical improvement in less time.

On the contrary, the results of this study highlight how a late diagnosis can lead to repeated infiltrative treatments, associating hydrodistention of the glenohumeral joint with the prolongation of physiotherapy for many months.

This study aims to highlight how ultrasound-guided hydrodistension and physiotherapy are an indispensable treatment for adhesive capsulitis, but early diagnosis is the key element for reducing time of treatment and pain, obtaining a better response to the infiltrative treatment associated to early rehabilitation, not acting on the histopathological nature of the pathology.

## Data Availability

The data that support the findings of this study are available from the corresponding author upon reasonable request.
